# Decay experiments and microbial community analysis of water lily leaf biofilms: Sediment effects on leaf preservation potential

**DOI:** 10.1371/journal.pone.0315656

**Published:** 2024-12-18

**Authors:** Brianne Palmer, Sabina Karačić, Shook Ling Low, Kathrin Janssen, Harald Färber, Moritz Liesegang, Gabriele Bierbaum, Carole T. Gee

**Affiliations:** 1 Bonn Organismic Institute of Biology, Division of Palaeontology, University of Bonn, Bonn, Germany; 2 Institute of Medical Microbiology, Immunology and Parasitology, University Hospital Bonn, Bonn, Germany; 3 Institute of Botany, Czech Academy of Sciences, Staré Město, Czech Republic; 4 Institute for Hygiene and Public Health, University Hospital Bonn, Bonn, Germany; 5 Institute of Geological Sciences, Department of Earth Sciences, Freie Universität Berlin, Berlin, Germany; Maria Curie-Sklodowska University: Uniwersytet Marii Curie-Sklodowskiej, POLAND

## Abstract

Understanding the intricate dynamics of sediment-mediated microbial interactions and their impact on plant tissue preservation is crucial for unraveling the complexities of leaf decay and preservation processes. To elucidate the earliest stages of leaf preservation, a series of decay experiments was carried out for three months on *Nymphaea* water lily leaves in aquariums with pond water and one of three distinctly different, sterilized, fine-grained substrates—commercially purchased kaolinite clay or fine sand, or natural pond mud. One aquarium contained only pond water as a control. We use 16S and ITS rRNA gene amplicon sequencing to identify and characterize the complex composition of the bacterial and fungal communities on leaves. Our results reveal that the pond mud substrate produces a unique community composition in the biofilms compared to other substrates. The mud substrate significantly influences microbial communities, as shown by the correlation between high concentrations of minerals in the water and bacterial abundance. Furthermore, more biofilm formers are observed on the leaves exposed to mud after two months, contrasting with declines on other substrates. The mud substrate also enhanced leaf tissue preservation compared to the other sediment types, providing insight into the role of sediment and biofilms in fossilization processes. Notably, leaves on kaolinite clay have the fewest biofilm formers by the end of the experiment. We also identify key biofilm-forming microbes associated with each substrate. The organic-rich mud substrate emerges as a hotspot for biofilm formers, showing that it promotes biofilm formation on leaves and may increase the preservation potential of leaves better than other substrates. The mud’s chemical composition, rich in minerals such as silica, iron, aluminum, and phosphate, may slow or suspend decay and facilitate biomineralization, thus paving the way toward leaf preservation. Our study bridges the information gap between biofilms observed on modern leaves and the mineral encrustation on fossil leaves by analyzing the microbial response in biofilms to substrate types in which fossil leaves are commonly found.

## Introduction

The fossil record provides a window into the history of life on Earth, offering us vignettes of past biodiversity, organismal evolution, and ecological dynamics. While hard tissues such as bones and shells have traditionally dominated paleontological research [1], many questions regarding the fossilization of soft tissues remain unanswered. Leaves, essential components of terrestrial ecosystems that consist of softer tissues compared to wood, also hold a wealth of information about paleoecology and climate [2]. However, the factors governing their fossilization, especially the earliest stages of leaf preservation, have remained elusive.

Decay experiments are an important approach that can be used to sort out biological and geological factors involved in fossilization. Among these factors, the role of substrate in preservation has received significant attention, because different sediment types can impact the preservation of organisms as varied as crustaceans [3], frogs [4], lobster eggs [5], and leaves [6]. Evidence of microbial mats found covering fossil organisms [7–9] and taphonomic observations and experiments on leaves [10–19] also suggest that protective biofilms produced by microbial activity may be an essential first stage for leaf tissue preservation. Since biofilms are primarily composed of water, it is likely that they will not fossilize. Consequently, there is no evidence that fossil plant material such as leaves without observable traces of biofilm were not originally covered by biofilms during the early stages of decay and subsequent preservation. However, the presence of organic-rich laminas such as those found in the Ediacaran of South Australia [20] and in the Cretaceous of Brazil [21, 22] indicate the potential role of biofilms in the fossilization of delicate structures as the biofilm may provide the appropriate taphonomic conditions to facilitate this preservation.

A biofilm is a structured community of microorganisms that forms on various surfaces, including living plant leaves [23]. It is composed of microorganisms, such as bacteria, fungi, and algae, embedded within a self-produced matrix of extracellular polymeric substances (EPS) [23]. The EPS matrix provides structural support and protection to the microorganisms within the biofilm and is made of polysaccharides, proteins, nucleic acids, and lipids [23]. Epiphytic microorganisms may harm the host plant by causing disease or through decomposition processes [24, 25], or benefit the plant by protecting it from pathogens and desiccation that may decay the plant structures [23].

Within the EPS matrix, the metabolism of many biofilm organisms can result in mineral production including calcium carbonate, iron, silica, and phosphate minerals. These minerals are deposited on and in the biofilm substrate through biomineralization [26–29]. The microorganisms can contribute minerals from their cell walls, such as the silica from diatom frustules, or influence the chemical conditions in their environment by metabolic processes, introducing pH shifts or alterations in the redox potential, that lead to precipitation. In addition, passive mineral precipitation can be induced by the negative charges of the biofilm polymers, which bind cations and serve as nucleation points for precipitation [30]. Such processes provide a mineral matrix that can reinforce and stabilize plant tissues during preservation [28] and could help prevent the degradation and disintegration of plant cells [31, 32]. Minerals commonly associated with plant fossilization include carbon, silica, calcium carbonate, iron, aluminum, and phosphate minerals [33].

Our goal is to describe the earliest stages of preservation and decay in water lily leaves through microbial biofilms. While other plant groups such as dicot leaves or fern pinnules have received some scientific attention in the lab [13, 16], the preservation potential of water lily leaves and the foliage of other water plants has remained a puzzle. Water lilies, in particular, have a long fossil history that extends to the advent of the angiosperms in the Early Cretaceous [34]. They were one of the first clades of flowering plants to evolve, appearing as early as 113 million years ago [35]. In the Cretaceous and Cenozoic record, the leaf is the most commonly preserved plant organ of the Nymphaeales and Nymphaeaceae [35].

Today, water lilies grow in low-energy, freshwater water environments, with fine-grained substrates, as they commonly did in the paleontological past (Table 1). These sorts of quiet lacustrine facies are particularly conducive to the preservation of fossil leaf compressions and impressions [15, 36]. However, water lilies are rarer in the fossil record than would be expected based on their habitat preferences in quiet freshwater environments [37, 38]. A *Nymphaea* leaf was recently described from the Miocene Clarkia leaf compression flora in Idaho, USA, which is widely known for its exceptional preservation [38]. Yet, despite the excellent fossilization and abundance of plant specimens collected from the Clarkia Lake deposit, up to now, four fossil leaves could be attributed to aquatic macrophytes, of which only one could be assigned to *Nymphaea* sp. [38]. Earlier research suggested that microbial mats, a form of complex oxygenic biofilm, might have generated microenvironments conducive to fossilization. However, varying oxygen and water levels in in freshwater lakes also play a significant role in the preservation process [39]. The rapid decay of water lily leaves through microbial degradation before and after deposition in the sediment or insect herbivory [40] certainly contributes to the underrepresentation of water lilies in the fossil record. However, if decay processes could be slowed down or halted by a protective, biofilm-facilitated, mineral coating on the leaf, then there would be a greater chance for the leaf to continue the pathway toward preservation instead of heading for biological degradation. In this way, the biofilm would act as a protective layer in the early taphonomic stages, allowing for the precipitation of minerals in later stages. This initial stage of biofilm protection is not understood, as prior research has focused mainly on later stages in the taphonomic process [16].

**Table 1:** Well-documented leaves of Nymphaeales or Nymphaeaceae in the fossil record. Here, Table 1 from Taylor and Gee (2014) is expanded by listing the lithology of the fossil matrix or fossil-bearing deposit, as well as the interpretation of the depositional environment of the fossil leaf site.

Taxon	Geologic age	Locality	Lithology of fossil matrix or fossil-bearing deposit	Interpretation of depositional environment	References
*Jaguariba wiersemana* Coiffard, Mohr et Bernardes-de-Oliveira	Early Cretaceous	Crato Formation, Brazil	Very fine-grained limestone	Shallow, lacustrine wetland	[41, 42]
*Pluricarpellatia petalta* Mohr, Bernardes-de-Oliveira et Taylor	Early Cretaceous	Crato Formation, Brazil	Very fine-grained limestone	Shallow, lacustrine wetland	[41, 42]
*Scutifolium jordanicum* Taylor, Brenner et Basha	Early Cretaceous	Jarash Formation, Jordan	Carbonized silt–clay bed	Possibly crevasse splay	[43]
*Aquatifolia fluitans* Wang et Dilcher	Cretaceous	Dakota Formation, Kansas, USA	Claystone	Shallow margin of a freshwater lake	[44, 45]
*Brasenites kansense* Wang et Dilcher	Early Cretaceous	Dakota Formation, Kansas, USA	Claystone	Shallow margin of a freshwater lake	[44, 45]
*Nymphaea* *mesozoica* Dobruskina	Late Cretaceous	Upper member of the Ora Shale Formation, Israel		Very shallow water environment, i.e., lake margin	[46]
Nymphaeaceous leaf similar to *Nuphar*	Late Cretaceous	Cantwell Formation, Alaska, USA	Fine-grained sandstone	Overbank sediments in shallow standing water	[47]
*Nuphar*? sp. 1	Late Cretaceous	Chorrillo Formation, Argentina	Dark, siliciclastic mudstone	Low-energy, paludal, or marginal freshwater environment	[48]
*Nuphaea engelhardtii* Gee et Taylor	Middle Eocene	Messel Formation, Germany	Extremely organic-rich shale*	Lake margin	[49, 50]
*Nymphaea elisabethae* Gee et Taylor	Late Oligocene	Rott Formation, Germany	Organic-rich siltstone*	Freshwater lake	[35, 51]
*Nymphaea lignitica* Wessel et Weber	Late Oligocene	Rott Formation, Germany	Organic-rich siltstone*	Freshwater lake	[35, 51, 52]
*Nymphaea* sp.	Middle Miocene	Clarkia local flora, Wanapum Formation, Idaho, USA	Light brown clay matrix in laminated lake sediments	Freshwater lake	[38, 53]

*Gee, own evaluation

To help unravel the complexities of sediment-mediated microbial interactions and their impact on soft tissue preservation, as well as to understand the puzzling paucity of water lily foliage in the fossil record of freshwater lakes, we selected the leaves of water lilies. Here, we conduct a multi-aquarium experiment in the laboratory using the leaves of *Nymphaea* sp. submerged in pond water with one of three distinct sediment types in which fossil leaves are commonly found—a clay-rich, organic-rich, or fine-sand substrate—along with a pond-water-only control. Amplicon sequencing of 16S and ITS rRNA is used to identify the bacteria and fungi comprising the biofilms and to characterize biofilm formers in the microbial communities on leaves in each of the substrate treatments over the course of three months.

## Methods

### Leaf collection and aquarium set-up

In July 2021, 35 green leaves of the water lily *Nymphaea* sp. were gathered from a large freshwater pond at the Bonn University Botanic Garden. The floating leaves were collected several meters away from shore using a rowboat. Upon collection, the leaves were immediately placed in the aquariums (S1 Fig). The pond is approximately 1.5 meters deep and fed primarily by a small stream and rainfall.

Pond water and mud from the pond bottom were also collected. The pond mud for the mud-substrate aquarium was sterilized before the start of the experiment. Pond water was collected in sterilized containers at the start of the experiment and immediately placed in the aquariums.

To determine the substrate minerology of each substrate, X-ray powder diffraction data were recorded on a PANalytical Empyrean diffractometer using CuKα radiation (λ = 1.54060 Å) at 40 kV and a tube current of 40 mA. Samples were hand-ground in an agate mortar and scanned on a rotating stage at 2°2θ/min (step size 0.02°2θ) from 5–80°2θ. The qualitative and quantitative mineralogical analysis was performed using the HighScore Plus PANalytical software. The pond mud contains a mineral composition of 30% quartz (SiO_2_), 62% illite ((K, H_3_O) (Al, Mg, Fe)_2_(Si, Al)_4_O_10_[(OH)_2_·(H_2_O)]), and 8% alkali feldspar (KAlSi_3_O_8_). Two other substrates were tested, kaolinite clay (Al_2_Si_2_O_5_(OH)_4_) and fine-grained quartz sand (SiO_2_), which were purchased commercially. The grain size of the substrates was not as factor in this experiment, but instead emphasized the differences in the mineral composition of the individual substrates.

The sediments were sterilized using an autoclave to ensure the starting microbial community was derived only from the leaves and pond water. The experiment was run in four 60 L aquaria. One aquarium contained pond water with no substrate as a control, while the other three aquariums contained pond water and one of the following fine-grained substrates: kaolinite clay, pond mud, or fine sand. Each aquarium was maintained at room temperature (~ 20°C) and was exposed to the natural daylight cycles in Bonn, which was 14 hours of daylight on average during the experiment.

Each water lily leaf was placed into a hand-sewn mesh pouch to ensure that all the tissue from individual leaves remained together during decay. Eight leaves were placed in each aquarium at the water–substrate interface, held down by some substrate along the edges of the pouches, but the leaves themselves were not allowed to overlap. Henceforth, for the sake of linguistic convenience, we will refer to the leaves in the various treatments as green leaves (leaves collected straight out of the pond in the Botanic Garden), control leaves (leaves in the aquarium with only pond water without any substrate), kaolinite clay leaves (leaves on the kaolinite clay substrate), mud leaves (leaves on the pond mud substrate), and sand leaves (leaves on the sand substrate).

### Sampling of aquarium leaves

Water lily leaves were sampled after one (T1), two (T2), or three (T3) months. Three additional leaves were collected from the pond and used as the T0 control (S1 Fig). Due to the large size of the water lily leaves and the limited space in the aquariums, only two leaves could be sampled from each treatment at T3. As time progressed, the leaves sunk and settled directly on the substrate. By T3, the substrate coated the top and bottom of the leaf surfaces. After the leaves were collected, a small section of each leaf was preserved in formaldehyde-acetic-alcohol (FAA: 70% ethanol, 4% formaldehyde, 2% acetic acid) for subsequent microscopy. Another small section of the leaf was dried and pressed between newspapers to preserve the leaf in its degraded state for later study. The rest of the leaves were preserved in TE Buffer at -20°C until DNA extraction.

### Scanning electron microscopy

The FAA-preserved leaves were transferred into a 70% ethanol solution and subsequently dried using 22 cycles in a Leica EM CPD300 critical point dryer (CPD) at the Nees Institute for the Biodiversity of Plants (now part of the Bonn Institute of Organismic Biology), University of Bonn. The CPD leaves were mounted on aluminum stubs and coated with a thin layer of palladium. Scanning electron microscopy (SEM) was carried out using a Tescan Vega 4 LMU Scanning Electron Microscope at the Division of Paleontology, Institute of Geosciences (now part of the Bonn Institute of Organismic Biology), University of Bonn.

### Analysis of water chemistry

Concurrent with the sampling of leaves at T1 and T2, water samples were collected in 50 ml Falcon tubes for chemical analysis. The water was measured for concentrations of iron, manganese, silica, calcium, magnesium, sodium, and potassium, as well as for electrical conductivity, pH, and temperature. The composition and concentration of both cations and anions were measured using inductively coupled plasma mass spectrometry (Agilent ICP-MS 7700) and ion chromatography (Metrohm IC 930 Compact Flex 947 linked to a Professional UV/VIS Vario detector) at the Institute for Hygiene and Public Health (University Hospital Bonn).

### DNA extraction and sequencing

Using the leaves preserved in TE Buffer, leaf biofilms were separated from leaf surfaces with sonication in an ultrasound bath (Branson 1210 Ultrasonic Cleaner; Emerson, St. Louis, Missouri, USA) for 2 minutes. Subsequently, the samples were vacuum filtered using a 0.25-micrometer pore filter (mixed cellulose ester membrane; Berrytec GmbH, Grünwald, Germany). The filters with the biofilm microbes were then cut into small pieces and kept in 2 mL microcentrifuge tubes with TE buffer and stored at -20°C until further processing.

DNA extraction from the collected biofilm samples was performed using the FastDNA Spin Kit for Soil, following the manufacturer’s protocol. This particular kit had previously been confirmed to be efficient for biofilm DNA extraction from water lily leaves [17]. The elution step was carried out using 50 microliters of Dnase-free water. The concentration and quality of the extracted DNA were assessed using a NanoDrop spectrophotometer (Thermo Fisher Scientific, Waltham, Massachusetts, USA).

For microbial community analysis, the V4 variable region of the 16S rRNA gene was amplified using the primers 16s-515F (GTGCCAGCMGCCGCGGTAA) and 16s-806R (GGACTACVSGGGTATCTAAT) [54]. The ITS (Internal transcribed spacer) region was amplified using the primers ITS1F (CTTGGTCATTTAGAGGAAGTAA) and ITS2R (GCTGCGTTCTTCATCGATGC) [55]. This allowed for the quantification of both bacterial (16S) and fungal (ITS) microbial communities. The polymerase chain reaction (PCR) reaction was a single-step PCR with HotStarTaq Plus Master Mix Kit (QIAGEN). The PCR consisted of an initial denaturation step at 95°C for 5 minutes, followed by cycling at 95°C for 30 seconds, 53°C for 40 seconds, and 72°C for 1 minute for 30–35 cycles. A final elongation step was performed at 72°C for 10 minutes.

Paired-end sequencing (bTEFAP) was conducted by MR. DNA (Shallowater, Texas, USA) using the Illumina MiSeq sequencing platform, following the manufacturer’s guidelines. Raw sequence data were processed using QIIME2 with default parameters [56]. Sequence quality control, denoising, and chimera removal were performed using DADA2 to generate amplicon sequence variants (ASVs) [57].

Taxonomic classification of the 16S rRNA sequences was accomplished using the Silva 138 database [58], while the Unite database was used for the ITS sequence classification [59]. ASVs and taxonomy data derived from the QIIME2 and DADA2 pipelines were integrated using R version 4.2.1 [60].

For 16S sequences, additional classification was performed using GreenGenes2 [61], which was employed for phenotypic inference using BugBase [62]. The BugBase phenotypic determination approach was employed to identify potential biofilm-forming bacteria present on the water lily leaves.

### Microbial community analysis

ASV richness and Simpson’s evenness were calculated separately for bacteria (16S) and fungi (ITS) using “vegan” [63]. Each dataset (16S and ITS) was rarified to the lowest number of sequences in each dataset (43,836 and 14,654, respectively). Statistical differences in richness and evenness were calculated between substrate and time using ANOVAs. We used “ampvis2” to create heat maps of the most abundant bacteria and fungi genera [64]. Similarities in the ASV composition for bacteria and fungi were visualized using Venn Diagrams and the package “ggvenn” [65] We used the “anosim” function in “vegan” to visualize the ASV diversity within and between groups.

We used “microeco” to visualize the community composition of each substrate and time point using an non-metric multidimensional scaling (NMDS) plot and Bray-Curtis distances [66]. The statistical difference in microbial community composition was calculated using a PERMANOVA (the “adonis2” function in “vegan”). Pairwise comparisons between substrates and time were calculated using the “pairwise.adonis2.”

The correlation of the mineral content and other parameters of the water samples with microbial community composition was assessed using the “envfit” function in “vegan” and plotted using a dbRDA (distance-based redundancy analysis) in “microeco”. Only environmental vectors with a statistically significant correlation (P<0.05) were inclided. Correlations between the fifty most abundant genera based on DESeq2 differential abundance calculations and water chemistry were visualized using Pearson’s correlation plot in “microeco” with a significance cutoff of P = 0.05.

The influence of water chemistry, time, and substrate on the microbial community composition was evaluated using a variation partitioning analysis (VPA) within “vegan”. First, we created a correlation matrix with the water chemistry data and removed those minerals with collinearity greater than 0.8, which resulted in a model with Fe, Ca, and K (S1 Table). Hence, the VPA consisted of substrate, time, and Fe, Ca, and K. The data were transformed using the “Hellinger” method. The resulting VPA shows the percent of the variation in the microbial community composition that is explained by these three minerals. We then performed separate redundancy analyses (RDAs) with ANOVAs to identify the individual impacts of substrate, time, and water chemistry on community composition.

Finally, we used the BugBase data (16S only) to determine differences in the abundance of biofilm-formers that developed between substrates over time. BugBase uses an algorithm to predict functional pathways such as biofilm formation. We filtered the BugBase OTUs to only include those tagged as “Forms Biofilms” and calculated the richness of biofilm formers for each substrate and time point.

## Results

### SEM images show differences between substrate treatments

Immediately after removal from the aquarium, there was evident decay of the kaolinite and sand leaves at T3 (S1 Fig). However, the differences in the level of decay between leaf samples from each substrate over time became distinctly clear after SEM analysis and the study of the pressed leaves themselves.

**Fig 1:**
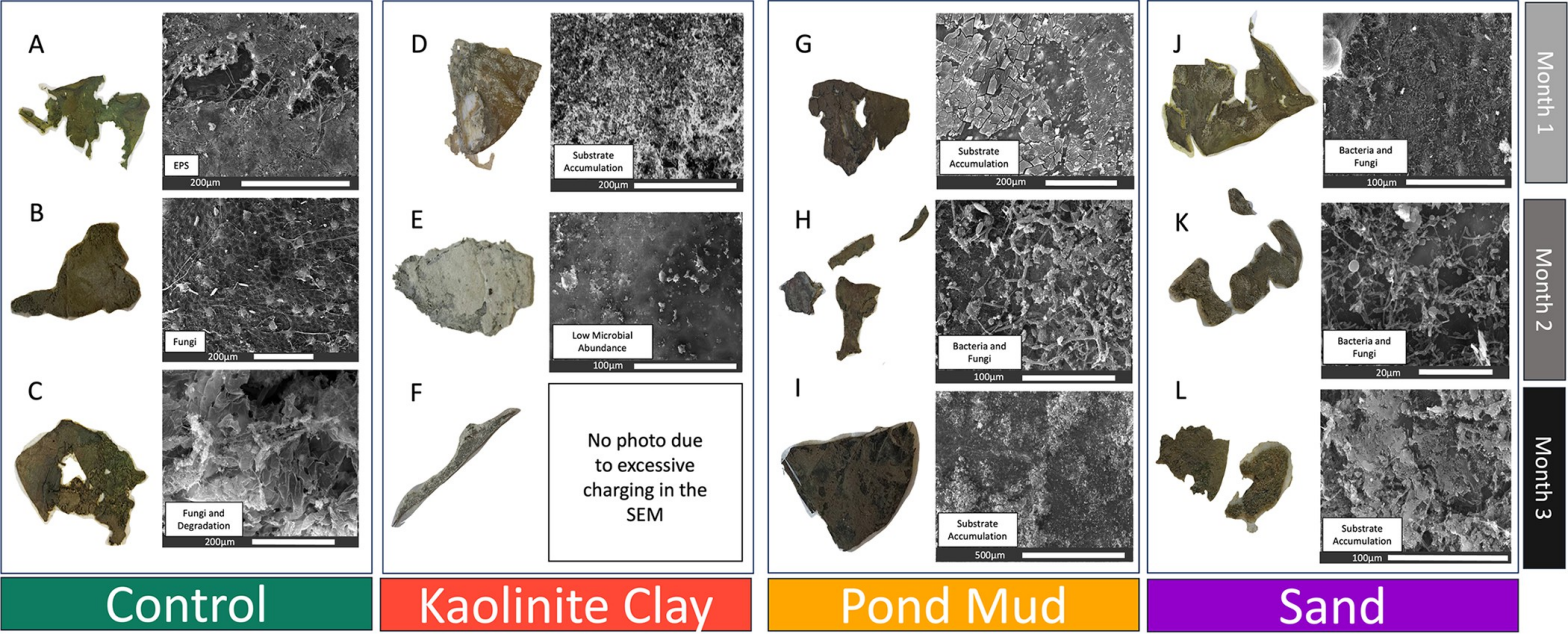
Photos and micrographs of water lily leaves over time. Conventional photos of leaves (left) and scanning electron micrographs of corresponding leaf surfaces (right). Leaves pressed in newspaper immediately after collection at all time points for the control (A–C), kaolinite clay (D–F), pond mud (G–I), and sand (J–L) treatments. Text boxes on the bottom corners of the micrographs describe the primary coverage of the leaf surface.

The control leaves at T1 were covered in bacteria and fungi. On the control leaves at T2, there was an increase in fungi, and microbially produced EPS could be observed. On the control leaves at T3, there were mineral particles, perhaps deriving from the pond water, on the leaf surfaces, and the leaves were coated in a biofilm with fungal hyphae. As observed on the pressed leaves, the leaf cuticles were still intact after 3 months (Fig 1). The pressed leaves consisted of pieces of leaf not preserved for SEM or used for DNA extraction and therefore do not necessarily represent the level of degradation of the full leaves.

The kaolinite clay leaves at all three time points were heavily coated in sediment, which made it difficult to discern any bacteria or fungi in the SEM images. When lifted out of the aquarium, all kaolinite clay leaves simply disintegrated when touched. The pressed leaves samples showed a thick layer of kaolinite clay, and little of the leaf structure was preserved (Fig 1).

The mud leaves at T1 had flakes of mud on their leaf surface, and there were visible remains of bacteria and fungi. The number of bacteria and fungi increased at T2, whereby the leaf surfaces were covered in fungal hyphae with bacteria embedded in the EPS between the fungi. At T3, the leaf surfaces were covered in sediment, much like the kaolinite clay treatment, and it was difficult to discern between bacteria and fungi. However, unlike the kaolinite clay samples, the mud leaves retained intact cuticles which had not completely disintegrated by the end of the experiment (Fig 1).

The sand leaves were covered in bacteria and fungi at T1, and like the leaves in the other sediments, the quantity of fungal filaments had increased by T2. At T3, fungi and EPS coated the leaf surface (Fig 1). The pressed sand leaves were not as degraded as the kaolinite clay leaves but showed more signs of decay compared to the mud and control leaves. Judging from only these visual observations, the sand leaves had also been colonized by microbial communities quite different from those on the mud, clay, and control leaves.

### Substrate type impacts microbial community composition

The 16S rRNA (bacteria and archaea) sequencing totaled 9,339,968 high-quality sequences, with an average of 490 ± 298 ASVs per sample. The ITS (fungi) sequencing yielded 2,574,143 high-quality sequences with an average of 126 ± 55 ASVs per sample.

**Fig 2:**
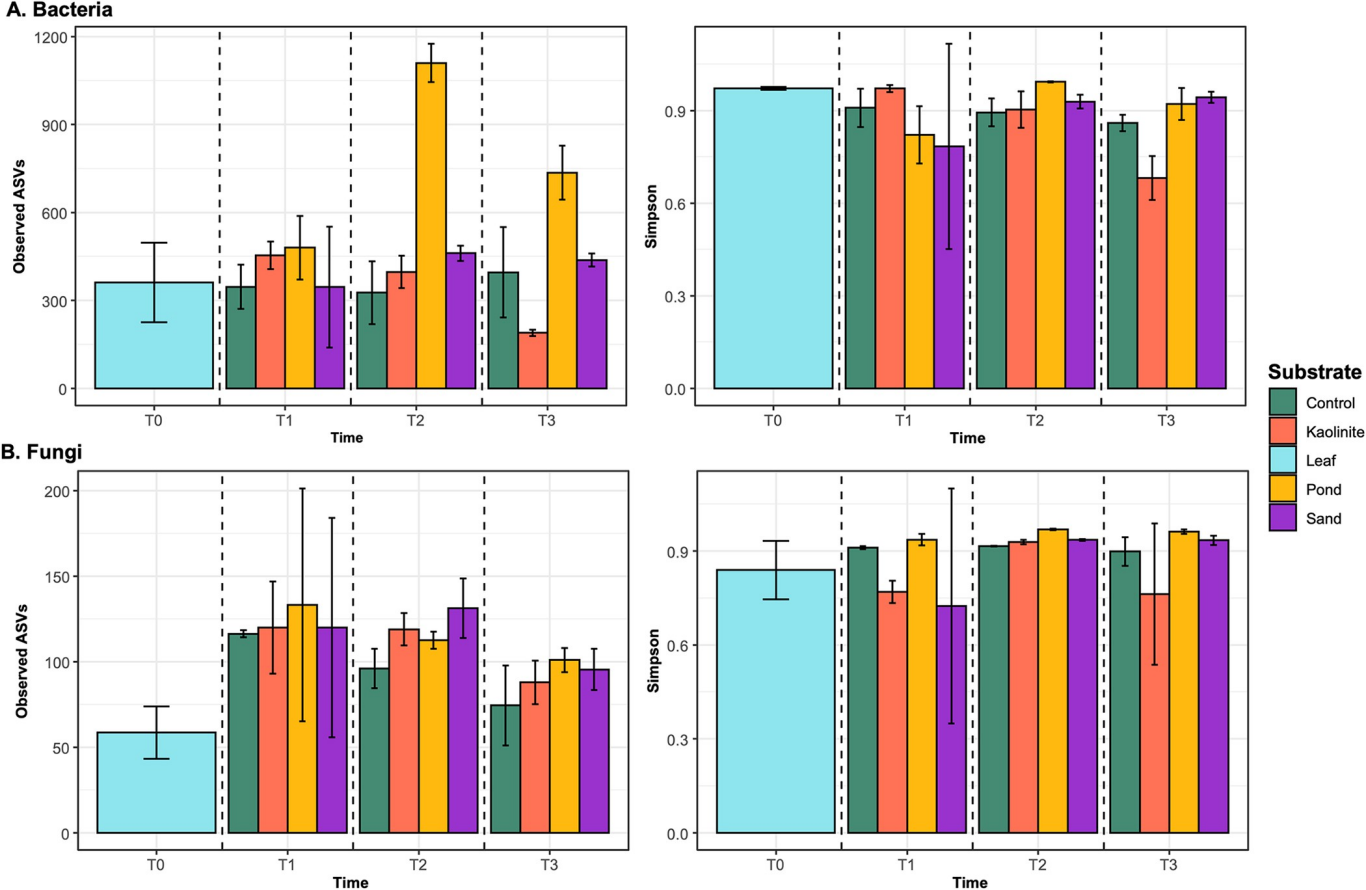
Richness and evenness of microbial communities. (Left) Richness of (A) bacteria and (B) fungi, and (right) Simpson’s evenness. The first column in blue represents the green leaf control at T0. The other sets of columns plot the values for the control (green) and each substrate (orange, yellow, purple) through time (T1, T2, T3). Dashed lines separate the time trials.

Regarding prokaryotic richness according to substrate type, the richness of the biofilm on the mud leaves was greater (1,576 ASVs) compared to that of leaves of all other substrates. The biofilm richness changed over time, peaking at T2 (Fig 2 and S2 Table). In contrast, Simpson’s evenness remained unchanged between substrates and over time (Fig 2 and S2 Table).

Fungal richness was significantly lower on the green leaves, compared to the mud and sand leaves (Fig 2 and S2 Table). Richness did not vary on the leaves on the other substrates. As with the bacteria, Simpson’s evenness remained constant in the leaf biofilms across all substrates throughout the experiment (Fig 2 and S2 Table).

Despite similarities in richness and evenness, the types of organisms colonizing the biofilms differed between substrates and over time. The most dominant bacterial phyla included Proteobacteria (44.8%), Firmicutes (19.8%), and Bacteroides (14.3%). It comes as no surprise, given its high richness value, that the biofilms of the mud leaves contained the greatest diversity of phyla (45), and that the low richness value of the green leaves meant that they had the fewest number (23). Ascomycota (12.7%) and Basidiomycota (4.9%) were the most dominant fungal phyla, although a large portion of the fungi could not be unidentified past the domain level (82%).

**Fig 3:**
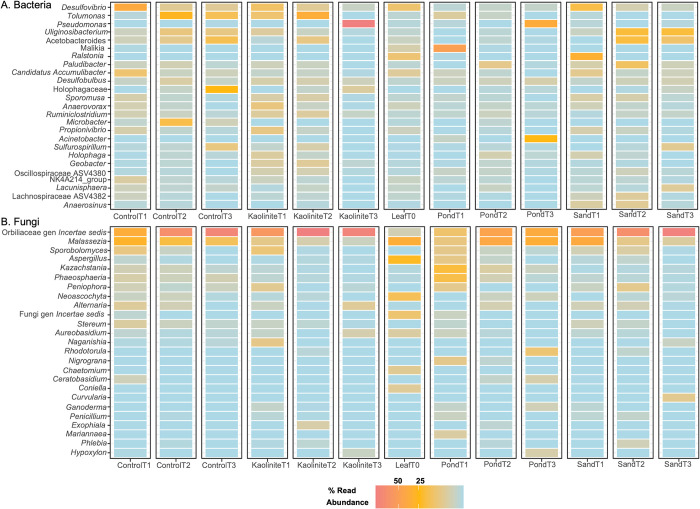
Heatmaps of genera abundance. Heatmaps for (A) bacterial and (B) fungal genera in each substrate at each time point. Red values indicate a high abundance, while blue values indicate a lower abundance. Each column represents one replicate.

By identifying the most dominant genera among substrates and in the course of time, we can start to understand the community composition and the ecological role of specific genera within the biofilms (Fig 3). *Desulfovibrio* was the most abundant genus; it was found primarily on the control, kaolinite clay, and sand leaves at T1 and decreased in abundance over time. *Pseudomonas* was very abundant on the kaolinite clay leaves at T3, comprising more than 50% of the read abundance. It was also found on the mud leaves at T3. It should be noted that there were few *Pseudomonas* sequences at the early time points. *Tolumonas* showed a parabolic trend and peaked in abundance at T2 on both the control and kaolinite clay leaves. *Malikia* was most dominant on the mud leaves at T1, while *Ralstonia* was more abundant on the sand leaves at T1.

As stated previously, most of the fungi could not be identified at the generic level. However, among those that could be assigned to a genus, *Malassezia* was common on the green leaves, control leaves, and mud leaves. *Sporobolomyces* was abundant across samples at T1. Several genera were found primarily on the green leaves, including *Aspergillus*, *Neoascochyta*, *Chaetomium*, and *Coniella*. The genera *Kazachstania* and *Phaeosphaeria* were abundant on one mud leaf.

**Fig 4:**
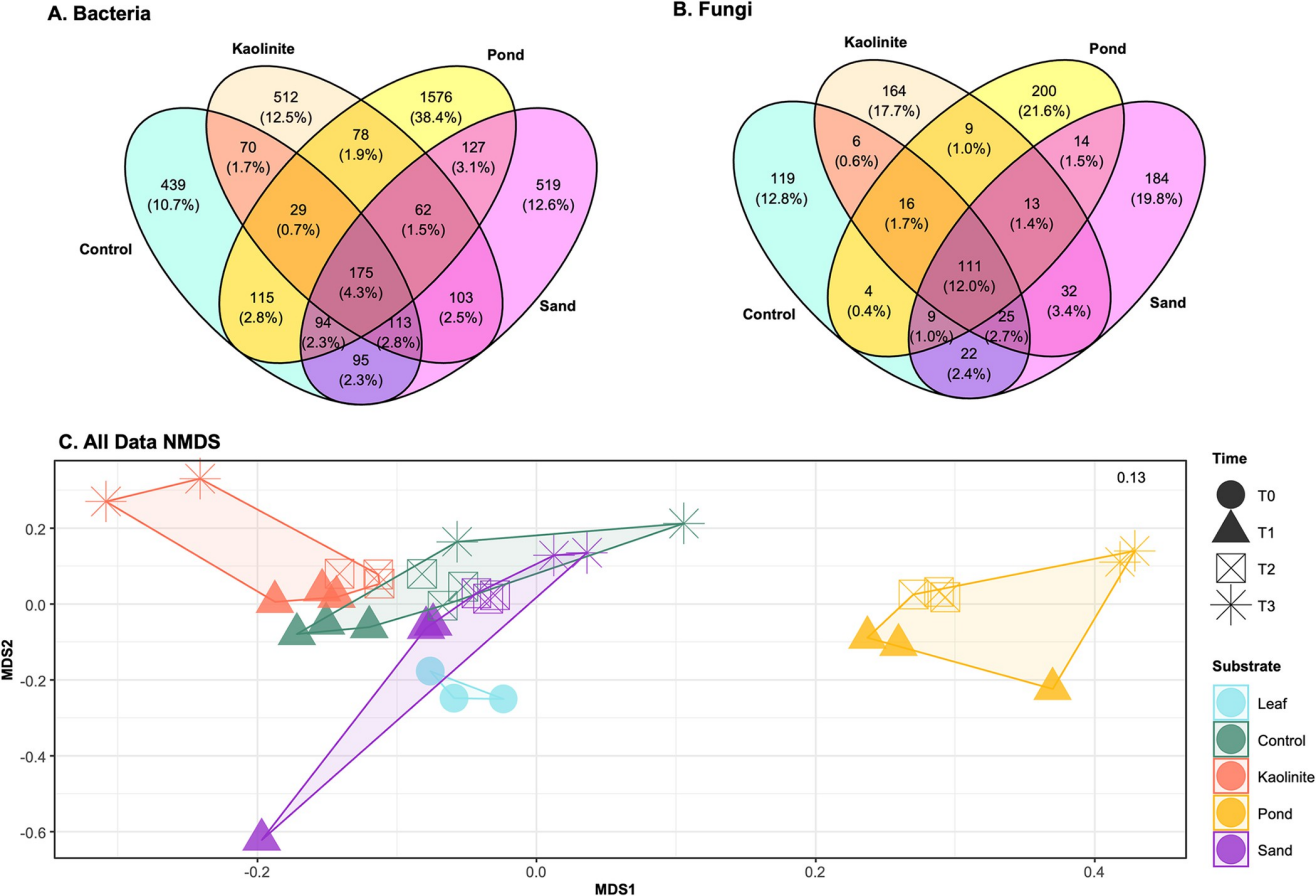
Venn diagrams of shared ASVs. Venn diagrams showing the number of ASVs shared between each substrate for (A) bacteria and (B) fungi. Sediment type is denoted by oval color: control (green), kaolinite clay (pink), pond mud (yellow), and sand (purple). (C) NMDS plot representing the influence of substrate (colors) and time (shapes) on microbial community composition. The polygons show the connections between samples within the same substrate at all time points. The stress value is located in the top right corner.

Given the differences in biofilm richness of the mud leaves compared to all that of leaves on all other substrates, as well as the variety of genera found in all leaves, it was not unexpected to find that community composition at each time point and across all substrates is unique (Fig 4). Only 4.3% of the bacterial ASVs were found in every substrate (excluding green leaf), compared to 12% of the fungal ASVs shared between substrates (Fig 4). The biofilm of the mud leaves had a more distinctive community composition at every time point, compared to biofilms on leaves on the other substrates. Based on the percentages of shared ASVs and the NMDS plot, the biofilms on the sand and control leaves had similar microbial communities, while those of the mud and kaolinite clay leaves were the most distinct from the green leaf. Based on the ANOSIM, there was a difference in the community composition between experimental conditions involving both substrate and time, although there was large variability in the community composition of a replication sand leaf at T1 which was considered during the interpretation here (S2 Fig). This variability was also evident in the NMDS plot (Fig 4 and S3 Table).

### Water chemistry and substrate interact and influence microbial community

**Fig 5:**
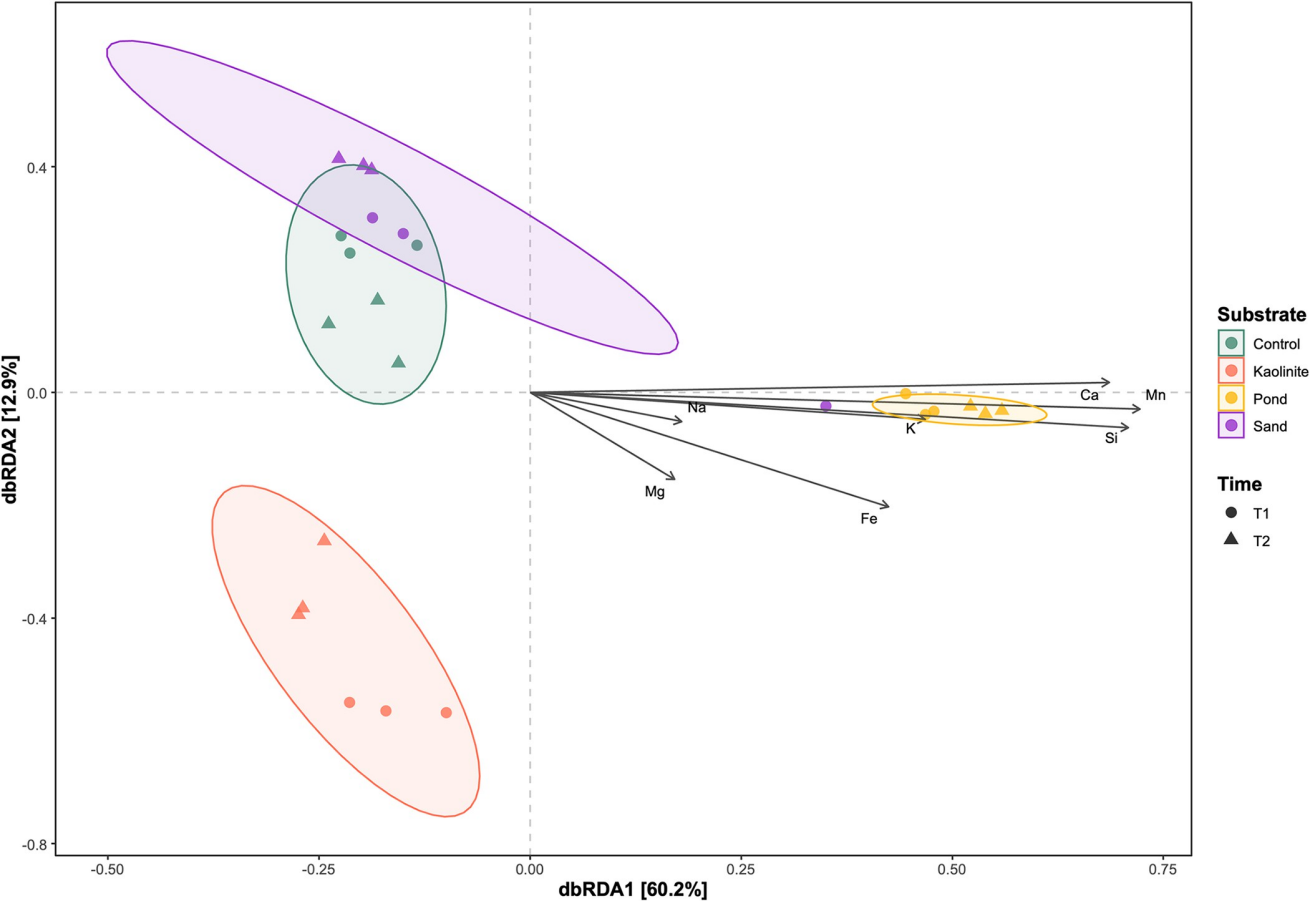
Ordination plot including water chemistry. A dbRDA depicting the correlation between water chemistry elements and microbial community composition. Each point represents one sample, which are grouped by substrate (color). A dot or triangle denotes time point T1 or T2, respectively.

The substrate in each aquarium greatly influenced the microbial community on the leaves. Substrate type also impacted the water chemistry in each aquarium, since the substrate added a distinctive set of minerals to the original chemistry of the pond water. The concentration of the water minerals measured varied between substrates and over time. Fe and Mg concentrations increased in the aquarium water with the kaolinite clay and pond mud substrates over time compared to the concentrations in the aquariums with the control and sand substrates. Due to its complex mineralogy, the water in the mud aquarium had greater concentrations of Mn, Ca, Si, and K, as well as higher conductivity compared to the other substrates. Na, pH, and temperature were similar across all substrates (S4 Table). Fe concentration and temperature were higher at T1, while Mg, Na, K, and pH peaked at T2. Furthermore, the concentration of minerals in the water correlated with microbial community composition (Fig 5). They are significantly correlated at T1 and T2, with greater mineral concentrations correlated with the community composition on the mud leaves. Fe, Mn, Si, Ca, K, and conductivity were significantly correlated with the Bray-Curtis distances (Fig 5 and S5 Table).

**Fig 6:**
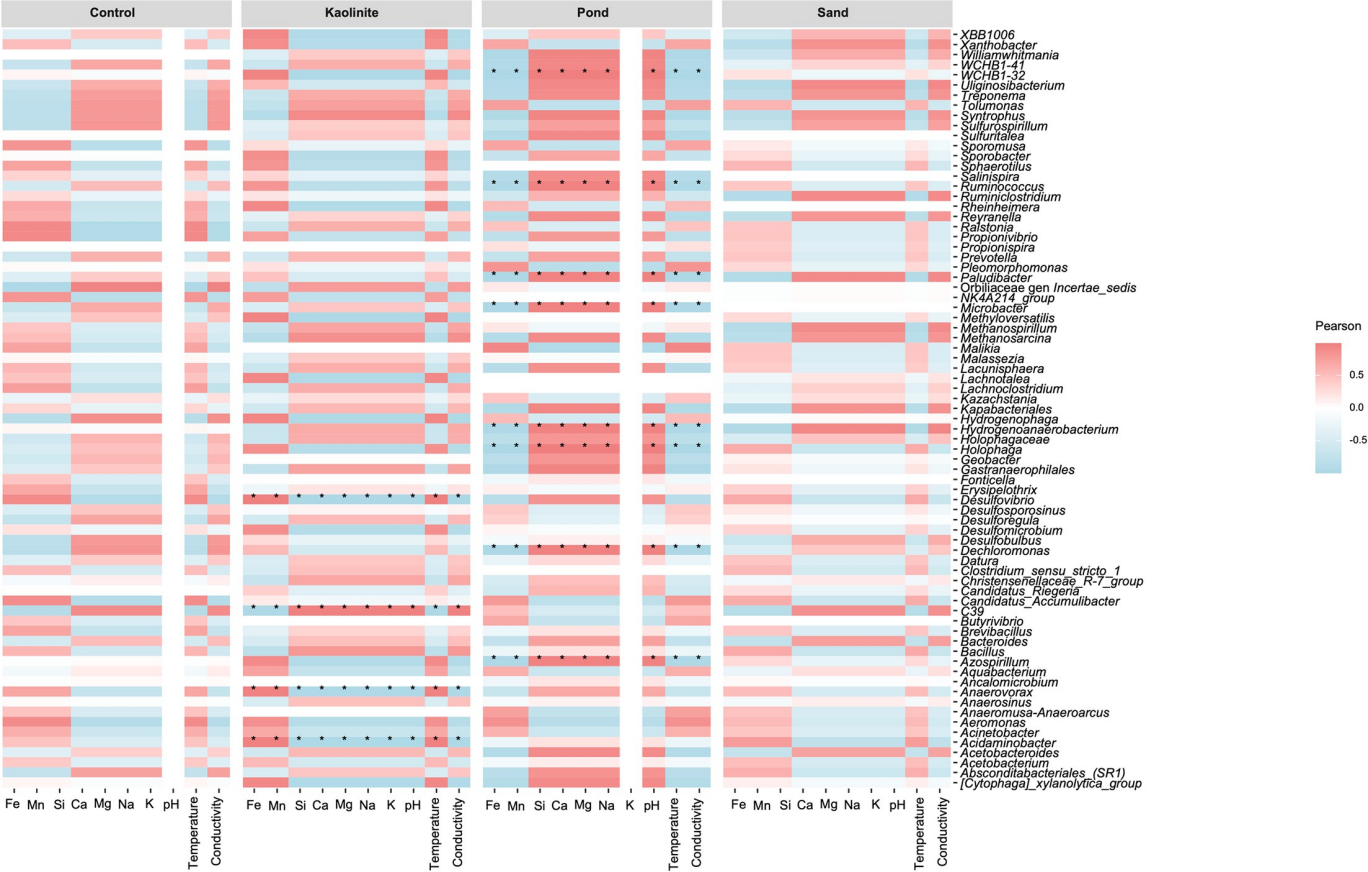
Correlation plot between water chemistry and genera. A heatmap showing Pearson’s correlations between the water chemistry elements and the most abundant genera, as grouped by substrate. Red indicates a positive correlation, while blue indicates a negative correlation. * denotes a p-value <0.05, ** p-value < 0.01, *** p-value < 0.001.

Likewise, several of the most abundant genera on the mud leaves were correlated with mineral concentration (Fig 6). All genera that were significantly correlated with the minerals in the mud substrate showed the same trend, aside from Na. All were negatively correlated with Fe, Mn, temperature, and conductivity, and all were positively correlated with Si, Ca, Mg, Na, and pH. These genera include WCHB1-32, *Ruminococcus*, *Microbacter*, *Holophaga*, *Dechloromonas*, and *Azospirillum*.

The abundant genera in the kaolinite showed the opposite trend. *Desulfovibrio Anaerovorax*, and *Acidaminobacter* were positively correlated with Si, Ca, Mg, Na, K, and pH, and were negatively correlated with Fe and Mn. Only Orbiliaceae gen. incertae sedis was significantly correlated with the concentrations of minerals in the control, and Na was correlated with those of the sand.

The VPA further confirms that substrate and water chemistry influence the microbial community composition (S3 Fig). Substrate alone best explains the variation in community composition between the groups (23.8%). The interaction between substrate and water chemistry (Fe, Si, and K) explains 18.3% of the variation, while water chemistry alone is responsible for 16.3% of the variation. Time produces the smallest effect (4.8%). However, 36.1% of the variation remains unexplained.

### Leaves on a mud substrate are a hot spot for biofilm-forming microbes

**Fig 7:**
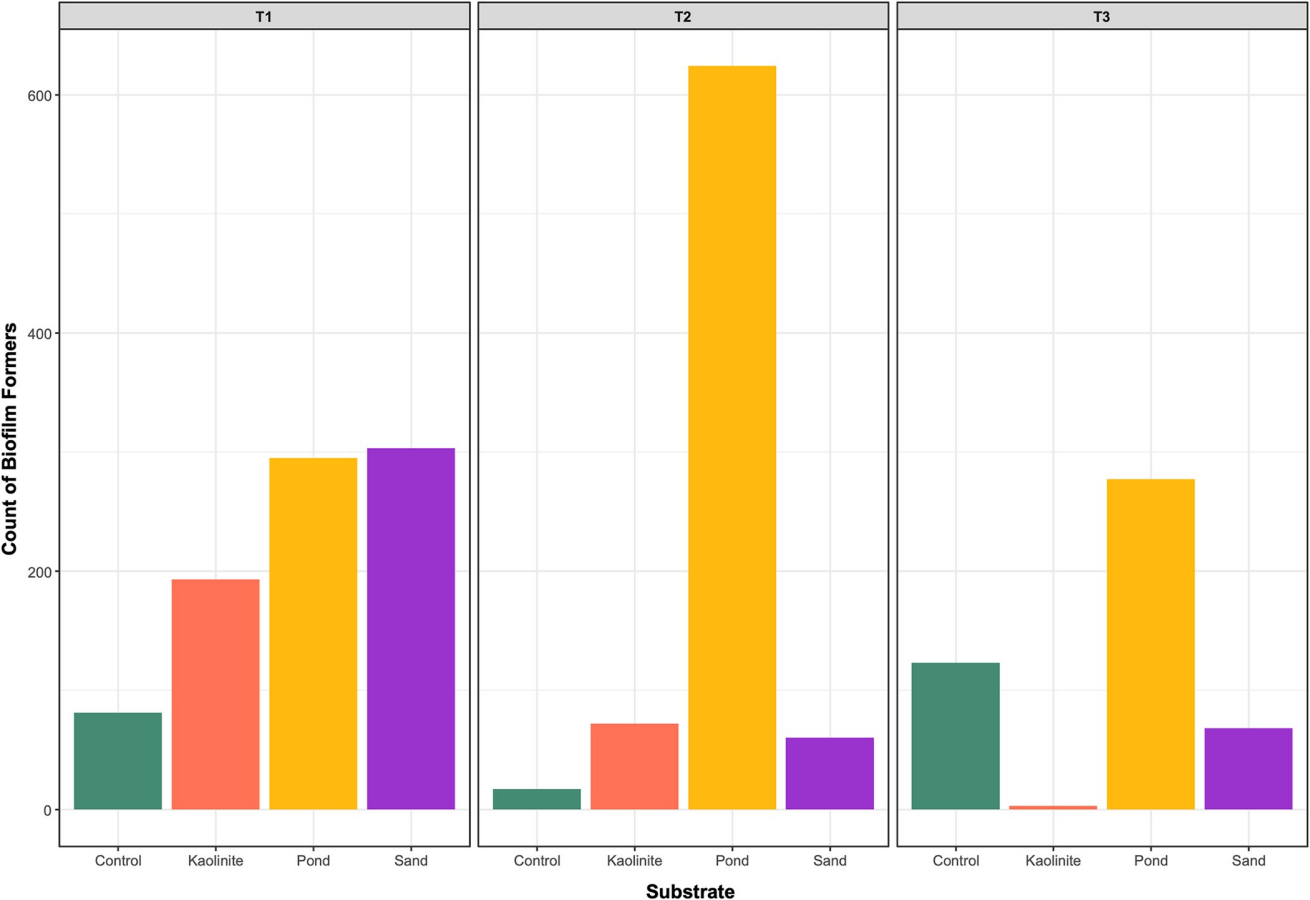
The abundance of biofilm-formers. The number of OTUs that were classified as biofilm formers by BugBase on the leaves collected from each sediment over time. The different colors denote the control treatment and three substrate treatments.

In addition to characterizing the microbial community composition and assembly processes, we investigated the potential role of biofilm-forming microorganisms in leaf preservation by counting the OTUs correlated with biofilm-producing bacteria in Bugbase. At T1, the control leaves contained the lowest number of biofilm formers, followed by the kaolinite clay, mud, and sand leaves. In Fig 1, biofilms are evident in the control leaves at T1, and based on the microbial analyses, these biofilms were formed by a low diversity of biofilm-formers. Notably, the number of biofilm formers doubled on the mud leaves at T2, while a decline was observed on leaves on the other substrates. The SEM images also show an increase in biofilm abundance in the mud at T2, though the presence of biofilms in T3 is obscured by the substrate coating the leaf surface (Fig 1). By T3, the kaolinite clay leaves had the lowest number of microbial biofilm formers among leaves on all substrates (Fig 7). In addition to having the lowest number of biofilm formers, there was little evidence of biofilms on the kaolinite leaves in the SEM images, regardless of the time point (Fig 1).

Over time, the genera of biofilm-forming microbes underwent notable shifts. OTUs in the fungal classes of Actinomycetes, Bacteroidia, Clostridia, and Spirochaetia primarily contributed to biofilm formation in the control leaves. At T2, only ASVs in the class Polyangia were identified as biofilm formers. By T3, members of the classes Polyangia, Ignavibacteria, and Spirochaetia contained biofilm-forming ASVs. On the kaolinite clay leaves, OTUs from Actinomycetes and Spirochaetia were identified as biofilm formers at T1, while Desulfobacteria, Gammaproteobacteria, Candidatus Kapabacteria, and Verrucomicrobiae contributed to biofilm formation at T2. Interestingly, only the phylum Candidatus Kapabacteria contained biofilm-forming ASVs at T3, albeit in low abundance.

In the mud leaves, biofilm formers were associated with the bacterial classes Alphaproteobacteria, Bacilli, and Clostridia at T1. By T2, Alphaproteobacteria, Bacilli, Bacteroidia, Clostridia, Gammaproteobacteria, Candidatus Kapabacteria, and Syntrophobacteria contained biofilm-forming ASVs. At T3, biofilm formers from the classes Bacilli, Bacteroidia, Clostridia, Gammaproteobacteria, Syntrophobacteria, and Verrucomicrobiae were identified.

Remarkably, the mud leaves had the highest number of bacterial biofilm formers. However, the class Actinomycetes, which was found in all other substrates, was conspicuously absent on all mud leaves. Moreover, the mud leaves uniquely hosted biofilm formers of the classes Alphaproteobacteria, Bacilli, Desulfobulbia, and Syntrophobacteria, underscoring their distinct microbial composition. Conversely, the control leaves stood out as the only ones with biofilm formers from the class Polyangia and no representatives of the Verrucomicrobiae (Fig 7).

## Discussion

### Decay patterns and the influence of sediment

Our investigation into microbial interactions and leaf preservation reveals that decay patterns and the microbial community composition of the biofilms on *Nymphaea* water lily leaves vary with sediment type and over time. Notably, the mud and control leaves exhibited the least decay over three months, while the kaolinite clay leaves underwent substantial decay. Furthermore, microbial community analysis reveals differences in the composition and assembly of microbial biofilms on each substrate over time.

Contrary to our results, previous research with animal carcasses found that carcasses buried in kaolinite had better preservation [67, 68], perhaps resulting from low bacterial abundance and diversity due to the interactions between the microbial community and the clay minerals, that resulted in lower bacterial abundance and diversity [68, 69]. However, similar to our results, a study with scallop carcasses buried in kaolinite indicates that burial in kaolinite leads to decay as muscles in kaolinite lost more mass compared to those buried in sand [70]. This strongly suggests that the sediment interactions, and consequently interactions with the microbial community, vary between organisms and potentially lead to substrate-selective preservation. Thus, it is important to understand the microbial community dynamics for individual groups of organisms.

The variations in bacterial genera across the different substrates and time points underscore the nuanced response of freshwater microbes to changing environmental conditions. *Pseudomonas*, while it appeared later, was abundant in the kaolinite clay and mud leaves, indicating its involvement in advanced stages of tissue decay. It is well-known that some strains of *Pseudomonas* are plant pathogens and can cause cankers, leaf and stem spots, blight, soft rot, and galls [71]. A previous experimental taphonomic study reported that *Pseudomonas* will form a biofilm around marine embryos, and while the bacteria will then consume the organic matter, the biofilm will retain the shape of the embryo [72].

Furthermore, some species of *Pseudomonas* can also form biofilms that replicate the external morphology and internal structure of embryos [73]. The appearance of *Pseudomonas* in the second month of our experimental decay series suggests that at this point, the environment provided the ideal conditions for *Pseudomonas* growth and may have prompted different responses from the original microbial community on the leaves. On the kaolinite leaves, *Pseudomonas* proliferated and became the most abundant genus in the biofilms. During this time, we also observe more decay on the kaolinite leaves compared to leaves on the other sediments. The biofilms on the mud leaves also contained *Pseudomonas*, though fewer ASVs. This indicates that although *Pseudomonas* was present on the mud leaves, the effect of decay may have been modulated by the high richness of other microbes on the leaves.

These other microbes include *Malikia*, *Tolumonas*, *Uliginosibacterium*, *and Ralstonia*, for example. *Malikia*, a biofilm former, was abundant on the mud leaves at T1. Elsewhere, it has been reported that *Malikia* performs well in aerobic environments [74]. *Tolumonas*, which was most common bacterium on the control and kaolinite clay leaves at T2, can be facultatively anaerobic and grow in anoxic freshwater [75, 76]. *Uliginosibacterium* was particularly abundant on the control and sand leaves at T2 and T3 and has previously been described from freshwater environments, such as sediment [77, 78] and lakes [79, 80]. The most abundant genus on the green leaves (but not in the aquariums) was Ralstonia, a genus known to cause plant diseases [81, 82]. That the bacterial communities were so variable between the sediments indicates that each sediment type attracted a differently adapted bacterial community.

Fungal communities exhibited different patterns over time and between sediment types, with *Malassezia* prevalent on green, control, and mud leaves. *Malassezia* is a pathogenic genus often found on the skin of animals [83]. The pond water used in our experiment came from a botanic garden freely open to the public, which may have been the source of the variety of human pathogens found growing on the leaves in the aquariums. In the first month, *Sporobolomyces* emerged as an abundant genus across all samples. It is a yeast that produces a red carotenoid pigment that has been shown to exhibit antimicrobial properties [84]. Furthermore, *Sporobolomyces* inhabits the phyllosphere [85] and may have come from within the leaf, instead of originating from the pond water. Other genera, namely, *Aspergillus*, *Neoascochyta*, *Chaetomium*, and *Coniella*, were found predominately on the green leaves, highlighting the diversity and abundance of fungi from the phyllosphere that may, in turn, impact the biofilm community during leaf decay.

Interestingly, the influence of elemental concentrations, particularly Fe, Mn, Si, Ca, K, and conductivity, on microbial community composition was significant, emphasizing the intricate relationship between environmental factors and microbial dynamics. Based on the correlation heatmap between water chemistry and the microbial community composition, communities on both the mud and kaolinite clay leaves correlated with several minerals in the water. However, these correlations were not necessarily the result of the ion concentrations but rather may have been influenced by the nutrients and organic matter present in the substrates. The pond mud was from a natural environment and may have contained more organic matter and nutrients that promoted the growth of different microbial communities, while the commercially purchased sand and kaolinite may have contained less naturally occurring organic matter and nutrients.

On the mud leaves, a correlation exists between the presence of *Paludibacter*, *Microbacter*, *Holophaga*, *Dechloromonas*, and *Azospirillum* with all minerals: there is a positive correlation with Si, Ca, Mg, Na, and pH, and a negative correlation with Fe, Mn, temperature, and conductivity. *Paludibacter*, *Microbacter*, and *Holophaga*, which were slightly more abundant on the mud leaves at T2, are anaerobic bacteria that excrete propionate and acetate [86–88]. *Dechloromonas* is a (per)chlorate-reducing genus [89], while *Azospirillum* is a microaerophilic, non-fermentative, and nitrogen-fixing genus [90]. The genera that correlate with the minerals in the water suggest that the environment on and within the biofilms was conducive to fermentation and anoxic lifestyles. Elsewhere, it has been noted that the excretion of acids may facilitate mineralization and aid in the preservation of soft tissues [91, 92].

On the kaolinite clay leaves, *Desulfovibrio*, *Anaerovorax*, *and Acinetobacter* were all positively correlated with Fe, Mn, and temperature. The dominance of *Desulfovibrio*, a sulfate-reducing bacterium, on the control, sand, and kaolinite clay leaves suggests these substrates provided an environment for Fe precipitation in the form of iron–sulfide solids (FeS) [93]. Previous studies have hypothesized that iron precipitation is a key process for leaf preservation, based on the observation of iron-encrusted plant fossils [6, 13]. However, the mud leaves—which had the least visible decay after three months—had lesser amounts of *Desulfovibrio*. *Anaerovorax* is strictly anaerobic bacterium with a fermentative metabolism, which excretes acetate and butyrate [94]. The genus *Acinetobacter*, on the other hand, can readily form biofilms [95] and furthermore can oxidize magnesium [96].

The application of the VPA further delineates the impact of substrate and water chemistry on microbial community composition. While the type of substrate is the most important factor, the interaction between substrate and water chemistry substantially contributes to much of the variation between the experimental treatments. Surprisingly, the variable of time exerted a relatively minor effect on community composition, reflecting the resilience of microbial communities to temporal changes.

Our study also identifies key biofilm-forming microbial OTUs associated with each substrate. The biofilms on the kaolinite clay leaves, for example, contained the sulfate-reducing, biomineralizing biofilm formers of *Desulfobacteria* [97, 98]. These biofilms also contained *Candidatus Kapabacteria* which has been found in anoxic, high-sulfide, wetlands [99], as well as in microbial mats in hot springs [100], indicating their ability to tolerate hostile environments. However, based on our observations of the rapid decay of the kaolinite leaves, it appears that the biofilm microbial community does not form a protective layer on the leaves that would prevent decay and encourage the formation of a mineral veil in the early taphonomic stages.

On the other hand, the genus *Clostridia* was also a prominent biofilm former on the mud leaves. Previous taphonomic work found *Clostridia* in the biofilms on decaying crayfish and hypothesized that *Clostridium* may be involved in adipocere formation and, consequently, instrumental in the pyritization of soft tissue fossils [101]. In leaves with less evident decay, like those in the mud, it is likely a consortium of microbes will tip the scales from decay towards a protective layer.

The shifting prevalence of biofilm formers over time and among different substrate types further emphasizes their dynamic roles in soft tissue preservation. It is worth mentioning again that the leaves on the mud substrate emerged as a hotspot for biofilm formers in our decay experiments. This, in combination with our observations of less morphological decay on the pressed mud leaves, suggests the biofilm-forming microbes likely slowed down leaf decay. Hence, mud substrates appear to support water lily leaf preservation better by facilitating the stronger development of more microbially diverse biofilms than kaolinite or sand clay substrates. The microbial-mediated biofilms would also facilitate mineral precipitation on leaf surfaces that will enclose and protect the leaf tissue from abrasion and damage during transport and from invertebrate herbivory, as well as slow down decay from further bacterial activity and preserve the fine-scale morphological features of the plant tissue [12, 102].

### Limitations of the experimental design

Although this multi-aquarium experiment was successful in elucidating the role of sediments on the biofilm microbial community of water lily leaves, there are important limitations to consider. First, microbial communities are highly variable, and the community composition and the environmental conditions can change over time which could impact replicability. Future work should perform the experiment over multiple timeframes throughout the year to identify changes in the microbial community and the effect of the substrate. Second, the sand and kaolinite were purchased commercially for this experiment while the organic-rich pond mud was derived from a nearby pond. In order to improve applicability of this experiment to natural conditions, future work should use naturally derived substrates of varying mineral compositions. Furthermore, though this work focused on the microbial composition of the biofilms, future experiments should address how sediment impacts the mineral content within the biofilms, as this likely differs between the organic-rich pond mud and the other sediments. This would also allow future researchers to test the role of organic matter in leaf fossilization. Finally, this study emphasizes the role of biofilms and sediment in the preservation of water lily leaves. However, the underrepresentation of water lily foliage in the fossil record is due in large part to rapid decay [38]. Future experiments should be performed with other plant leaves abundant in the fossil record to identify if these patterns are consistent across plant species.

### The preservation of water lily leaves in the fossil record

Here, we provide evidence that biofilm formation and biofilm microbial communities differ depending on the substrate in the early stages of leaf decay. These differences in community composition and the consequential metabolisms of the microbes could slow decomposition long enough for the formation of a mineral veil that can protect the leaf until it enters the ideal conditions for fossilization [13]. This veil may not become preserved in the fossil record, but act as a protective barrier until the conditions for preservation are met. Thus, the occurrence of water lilies in the fossil record may be influenced by the substrate type and, consequently, the biofilm microbial community on their leaves during decay.

In our survey of well-documented occurrences of water lilies in the geological record (Table 1), the fossil leaves are nearly always found in sedimentary rocks such as marlstone, claystone, mudstone, and siltstone [103]. Common to most examples, too, is the occurrence of a dark, carbonaceous, or organic-rich rock matrix, which may represent the muddy substrate at the bottom of an ancient pond or lake.

The preservation of the fossil water lily leaves in such sediments does not come as much of a surprise, given the biofilm-enhancing properties of a pond mud substrate, which is attributed to the mud substrate’s rich and complex mineralogy. It is also perhaps not a coincidence that the site with the greatest number of water lily specimens, totaling over 39 leaves, bears the extremely dark, oil-bearing shales of the middle Eocene Lake Messel in Germany [50] and is the most organic-rich locality of all those described here with fossil Nymphaeales and Nymphaeaceae foliage.

Thus, the sedimentology of these water lily leaf-bearing sites implies that a substrate such as pond or lake mud may be the most conducive for the fossilization of nymphaealean leaves. As shown, a mud substrate containing a complex suite of minerals may support the preservation of leaf tissues better than other substrates by facilitating the stronger development of microbially diverse, biomineralizing biofilms. To produce well-preserved fossil leaves, the development of strong, protective biofilms in the earliest stages of leaf decay, before the onset of substantial tissue degradation, may be crucial. The need for a protective biofilm has also been emphasized in several other studies [15, 18].

## Conclusions

Decay experiments on *Nymphaea* water lily leaves in aquariums were carried out for three months to elucidate the effect of different fine-grained substrates on microbes and biofilms. Using 16S rRNA and ITS gene amplicon sequencing, it was revealed that microbial communities in the leaf biofilms were significantly influenced by the mud substrate, as was reflected in the correlation between bacterial abundance and high concentrations of minerals in the water. In contrast to the leaf biofilms in the aquariums with kaolinite clay, fine-grained sand, or no substrate at all (the control), more bacterial biofilm formers were found on the leaves on the mud substrate after two months. Thus, the organic and mineral-rich mud substrate emerges as a hotspot for biofilm formers, which may increase the preservation potential of leaves that are deposited in such an environment. Our survey of the fossil record shows there is an association between fossil water lily leaves and a fine-grained, usually dark, carbonaceous, or organic-rich matrix, which may represent the muddy substrate at the bottom of an ancient pond or lake. Hence, the sedimentology of the fossil sites also suggests that an organic-rich lake or pond mud is a substrate that is conducive for the fossilization of nymphaealean leaves.

## Supporting information

S1 Table Pearson correlation between the chosen water chemistry variables (Fe, Ca, and K). Variables that had collinearity with other chemistry variables >0.8 were removed.
(DOCX)

S2 Table Results from the TukeyHSD comparisons for richness and evenness of bacteria and fungi. Values in red indicate the p-value is < 0.05.
(DOCX)

S3 Table Results from the PERMANOVA (adonis2 function) showing the effect of substrate and time on the microbial community composition (bacteria and fungi combined). (DOCX)

S4 Table The total concentrations of the ions and anions measured in the water at T1 and T2 in each substrate. (DOCX)

S5 Table Permutation test for dbrda under reduced mode. Terms were added sequentially (first to last) with 999 permutations based on the distance-based RDA plot. Only variables that had a significant p-value (<0.05) are shown.
(DOCX)

S1 Fig The aquarium set up with A) T1, B) T2, and C) T3. Each row contains the control, kaolinite, pond mud, and sand aquariums. C) depicts the leaves out of the aquarium after the final collection. D) shows evident of the kaolinite and sand leaves.
(DOCX)

S2 Fig Results of the ANOSIM (analysis of similarities) for bacteria and fungi combined. The betweenness shows the difference in the percentage of dissimilarity between all samples within the groups with corresponding R and p-values. The other boxplots denote differences in the percentage of dissimilarity within the groups for each of the three replicates.
(DOCX)

S3 Fig Visualization of the results of the VPA analysis. Each circle represents substrate, time, or water chemistry (Fe+Ca+K). The numbers within the circles show the fraction of variation explained by each.
(DOCX)
