# Supplementary material for: Decay experiments and microbial community analysis of water lily leaf biofilms: Sediment effects on leaf preservation potential
Source: PLoS One. 2024 Dec 18;19(12):e0315656. doi: 10.1371/journal.pone.0315656 (PMC11654923; doi:10.1371/journal.pone.0315656)
Supplement: S1 Table — Variables that had collinearity with other chemistry variables >0.8 were removed. (DOCX) [file pone.0315656.s001.docx]

Table S1: Pearson correlation between the chosen water chemistry variables (Fe, Ca, and K). Variables that had collinearity with other chemistry variables >0.8 were removed.

|  | Fe | Ca | K |
| --- | --- | --- | --- |
| Fe | 1 | 0.3762675 | 0.3181118 |
| Ca | 0.3762675 | 1 | 0.773168 |
| K | 0.3181118 | 0.773168 | 1 |
