# Supplementary material for: Decay experiments and microbial community analysis of water lily leaf biofilms: Sediment effects on leaf preservation potential
Source: PLoS One. 2024 Dec 18;19(12):e0315656. doi: 10.1371/journal.pone.0315656 (PMC11654923; doi:10.1371/journal.pone.0315656)
Supplement: S2 Table — Values in red indicate the p-value is < 0.05. (DOCX) [file pone.0315656.s002.docx]

Table S2: Results from the TukeyHSD comparisons for richness and evenness of bacteria and fungi. Values in red indicate the p-value is < 0.05.

|  | Richness | | | | Evenness | | | |  |
| --- | --- | --- | --- | --- | --- | --- | --- | --- | --- |
|  | diff | lwr | upr | p-adj | diff | lwr | upr | p-adj | Bacteria |
| kaolinite-control | 15.125 | -225.776 | 256.026 | 1.000 | -0.018 | -0.197 | 0.162 | 0.998 |  |
| leaf-control | 10.167 | -316.015 | 336.348 | 1.000 | 0.081 | -0.163 | 0.324 | 0.868 |  |
| mud-control | 428.625 | 187.724 | 669.526 | 0.000 | 0.019 | -0.160 | 0.199 | 0.998 |  |
| sand-control | 60.500 | -180.401 | 301.401 | 0.947 | -0.013 | -0.193 | 0.167 | 0.999 |  |
| leaf-kaolinite | -4.958 | -331.140 | 321.223 | 1.000 | 0.098 | -0.145 | 0.342 | 0.764 |  |
| mud-kaolinite | 413.500 | 172.599 | 654.401 | 0.000 | 0.037 | -0.143 | 0.217 | 0.974 |  |
| sand-kaolinite | 45.375 | -195.526 | 286.276 | 0.981 | 0.004 | -0.175 | 0.184 | 1.000 |  |
| mud-leaf | 418.458 | 92.277 | 744.640 | 0.007 | -0.061 | -0.305 | 0.182 | 0.947 |  |
| sand-leaf | 50.333 | -275.848 | 376.515 | 0.991 | -0.094 | -0.337 | 0.149 | 0.792 |  |
| sand-mud | -368.125 | -609.026 | -127.224 | 0.001 | -0.033 | -0.213 | 0.147 | 0.983 |  |
| T1-T0 | -71.063 | -362.511 | 220.386 | 0.909 | -0.017 | -0.234 | 0.201 | 0.996 |  |
| T2-T0 | 96.188 | -195.261 | 387.636 | 0.804 | 0.042 | -0.176 | 0.259 | 0.953 |  |
| T3-T0 | -37.688 | -343.361 | 267.986 | 0.987 | -0.037 | -0.265 | 0.191 | 0.971 |  |
| T2-T1 | 167.250 | -17.078 | 351.578 | 0.086 | 0.059 | -0.079 | 0.196 | 0.655 |  |
| T3-T1 | 33.375 | -172.710 | 239.460 | 0.971 | -0.020 | -0.174 | 0.134 | 0.984 |  |
| T3-T2 | -133.875 | -339.960 | 72.210 | 0.307 | -0.079 | -0.232 | 0.075 | 0.513 |  |
| kaolinite-control | 13.375 | -28.006 | 54.756 | 0.878 | -0.082 | -0.262 | 0.098 | 0.679 | Fungi |
| leaf-control | -39.583 | -95.614 | 16.447 | 0.266 | -0.070 | -0.314 | 0.174 | 0.917 |  |
| mud-control | 19.250 | -22.131 | 60.631 | 0.660 | 0.046 | -0.135 | 0.226 | 0.946 |  |
| sand-control | 19.875 | -21.506 | 61.256 | 0.633 | -0.053 | -0.234 | 0.127 | 0.908 |  |
| leaf-kaolinite | -52.958 | -108.989 | 3.072 | 0.071 | 0.012 | -0.232 | 0.256 | 1.000 |  |
| mud-kaolinite | 5.875 | -35.506 | 47.256 | 0.994 | 0.127 | -0.053 | 0.308 | 0.265 |  |
| sand-kaolinite | 6.500 | -34.881 | 47.881 | 0.990 | 0.029 | -0.152 | 0.209 | 0.990 |  |
| mud-leaf | 58.833 | 2.803 | 114.864 | 0.036 | 0.116 | -0.128 | 0.360 | 0.644 |  |
| sand-leaf | 59.458 | 3.428 | 115.489 | 0.033 | 0.017 | -0.227 | 0.261 | 1.000 |  |
| sand-mud | 0.625 | -40.756 | 42.006 | 1.000 | -0.099 | -0.279 | 0.081 | 0.510 |  |
| T1-T0 | 11.042 | -39.022 | 61.106 | 0.931 | -0.052 | -0.270 | 0.166 | 0.915 |  |
| T2-T0 | 3.375 | -46.689 | 53.439 | 0.998 | 0.050 | -0.168 | 0.268 | 0.921 |  |
| T3-T0 | -21.625 | -74.132 | 30.882 | 0.678 | 0.002 | -0.226 | 0.231 | 1.000 |  |
| T2-T1 | -7.667 | -39.330 | 23.997 | 0.911 | 0.102 | -0.036 | 0.240 | 0.204 |  |
| T3-T1 | -32.667 | -68.067 | 2.734 | 0.079 | 0.054 | -0.100 | 0.208 | 0.774 |  |
| T3-T2 | -25.000 | -60.401 | 10.401 | 0.240 | -0.048 | -0.202 | 0.106 | 0.829 |  |
