# Supplementary material for: Decay experiments and microbial community analysis of water lily leaf biofilms: Sediment effects on leaf preservation potential
Source: PLoS One. 2024 Dec 18;19(12):e0315656. doi: 10.1371/journal.pone.0315656 (PMC11654923; doi:10.1371/journal.pone.0315656)
Supplement: S3 Table — (DOCX) [file pone.0315656.s003.docx]

Table S3: Results from the PERMANOVA (adonis2 function) showing the effect of substrate and time on the microbial community composition (bacteria and fungi combined).

|  | Df | SumOfSqs | R2 | F | Pr(>F) |
| --- | --- | --- | --- | --- | --- |
| Substrate | 4 | 4.6478 | 0.39827 | 9.352 | 0.001 |
| Time | 2 | 1.4451 | 0.12383 | 5.8154 | 0.001 |
| Substrate:Time | 6 | 2.8438 | 0.24368 | 3.8147 | 0.001 |
| Residual | 22 | 2.7334 | 0.23422 |  |  |
| Total | 34 | 11.6702 | 1 |  |  |
