# Supplementary material for: Decay experiments and microbial community analysis of water lily leaf biofilms: Sediment effects on leaf preservation potential
Source: PLoS One. 2024 Dec 18;19(12):e0315656. doi: 10.1371/journal.pone.0315656 (PMC11654923; doi:10.1371/journal.pone.0315656)
Supplement: S4 Table — (DOCX) [file pone.0315656.s004.docx]

Table S4: The total concentrations of the ions and anions measured in the water at T1 and T2 in each substrate.

| Time | Substrate | Date | Fe (mg/L) | Mn (mg/L) | Si (mg/L) | Ca (mg/L) | Mg (mg/L) | Na (mg/L) | K (mg/L) | pH (mg/L) | Temp °C | Conductivity (μS/cm) |
| --- | --- | --- | --- | --- | --- | --- | --- | --- | --- | --- | --- | --- |
| T1 | control | 29.07.21 | 0.02 | 0.036 | 4.5 | 77 | 20 | 31 | 6.8 | 7.7 | 22.7 | 702 |
| T1 | kaolinite | 29.07.21 | 0.67 | 0.12 | 6.3 | 68 | 21 | 34 | 9.6 | 7.6 | 22.6 | 705 |
| T1 | mud | 29.07.21 | 0.62 | 1.1 | 16 | 114 | 22 | 37 | 14 | 7.9 | 22.7 | 949 |
| T1 | sand | 29.07.21 | 0.12 | 0.15 | 5 | 71 | 20 | 32 | 8.1 | 7.5 | 22.8 | 686 |
| T2 | control | 17.08.21 | 0.012 | 0.005 | 4.4 | 84 | 22 | 38 | 13 | 7.7 | 19.2 | 747 |
| T2 | kaolinite | 17.08.21 | 0.013 | 0.0053 | 6.5 | 76 | 24 | 39 | 11 | 8.2 | 21.3 | 726 |
| T2 | mud | 17.08.21 | 0.34 | 0.89 | 17 | 121 | 23 | 38 | 14 | 8.2 | 20.5 | 932 |
| T2 | sand | 17.08.21 | 0.058 | 0.005 | 4.9 | 76 | 22 | 39 | 11 | 7.8 | 21.1 | 753 |
