# Supplementary material for: Decay experiments and microbial community analysis of water lily leaf biofilms: Sediment effects on leaf preservation potential
Source: PLoS One. 2024 Dec 18;19(12):e0315656. doi: 10.1371/journal.pone.0315656 (PMC11654923; doi:10.1371/journal.pone.0315656)
Supplement: S5 Table — Terms were added sequentially (first to last) with 999 permutations based on the distance-based RDA plot. Only variables that had a significant p-value (<0.05) are shown. (DOCX) [file pone.0315656.s005.docx]

Table S5: Permutation test for dbrda under reduced mode. Terms were added sequentially (first to last) with 999 permutations based on the distance-based RDA plot. Only variables that had a significant p-value (<0.05) are shown.

|  | Df | SumOfSqs | F | Pr(>F) |
| --- | --- | --- | --- | --- |
| Mn | 1 | 1.86281 | 17.3565 | 0.001 |
| Ca | 1 | 0.71821 | 6.6918 | 0.001 |
| Na | 1 | 0.68502 | 6.3825 | 0.001 |
| Si | 1 | 0.62594 | 5.8321 | 0.001 |
| K | 1 | 0.4908 | 4.573 | 0.001 |
| Temp | 1 | 0.71832 | 6.6928 | 0.001 |
| Fe | 1 | 0.2724 | 2.5381 | 0.01 |
| Residual | 16 | 1.71723 |  |  |
