# Supplementary material for: Decay experiments and microbial community analysis of water lily leaf biofilms: Sediment effects on leaf preservation potential
Source: PLoS One. 2024 Dec 18;19(12):e0315656. doi: 10.1371/journal.pone.0315656 (PMC11654923; doi:10.1371/journal.pone.0315656)
Supplement: S1 Fig — The aquarium set up with A) T1, B) T2, and C) T3. Each row contains the control, kaolinite, pond mud, and sand aquariums. C) depicts the leaves out of the aquarium after the final collection. D) shows evident of the kaolinite and sand leaves. (DOCX) [file pone.0315656.s006.docx]

**Fig. S1:** The aquarium set up with A) T1, B) T2, and C) T3. Each row contains leaves from the control, kaolinite clay, pond mud, and sand aquariums, respectively. C) depicts the leaves taken out of the aquariums after the final collection. D) shows close-ups of the highly degraded condition of the kaolinite clay and sand leaves.
