# Supplementary material for: Decay experiments and microbial community analysis of water lily leaf biofilms: Sediment effects on leaf preservation potential
Source: PLoS One. 2024 Dec 18;19(12):e0315656. doi: 10.1371/journal.pone.0315656 (PMC11654923; doi:10.1371/journal.pone.0315656)
Supplement: S2 Fig — The betweenness shows the difference in the percentage of dissimilarity between all samples within the groups with corresponding R and p-values. The other boxplots denote differences in the percentage of dissimilarity within the groups for each of the three replicates. (DOCX) [file pone.0315656.s007.docx]

**
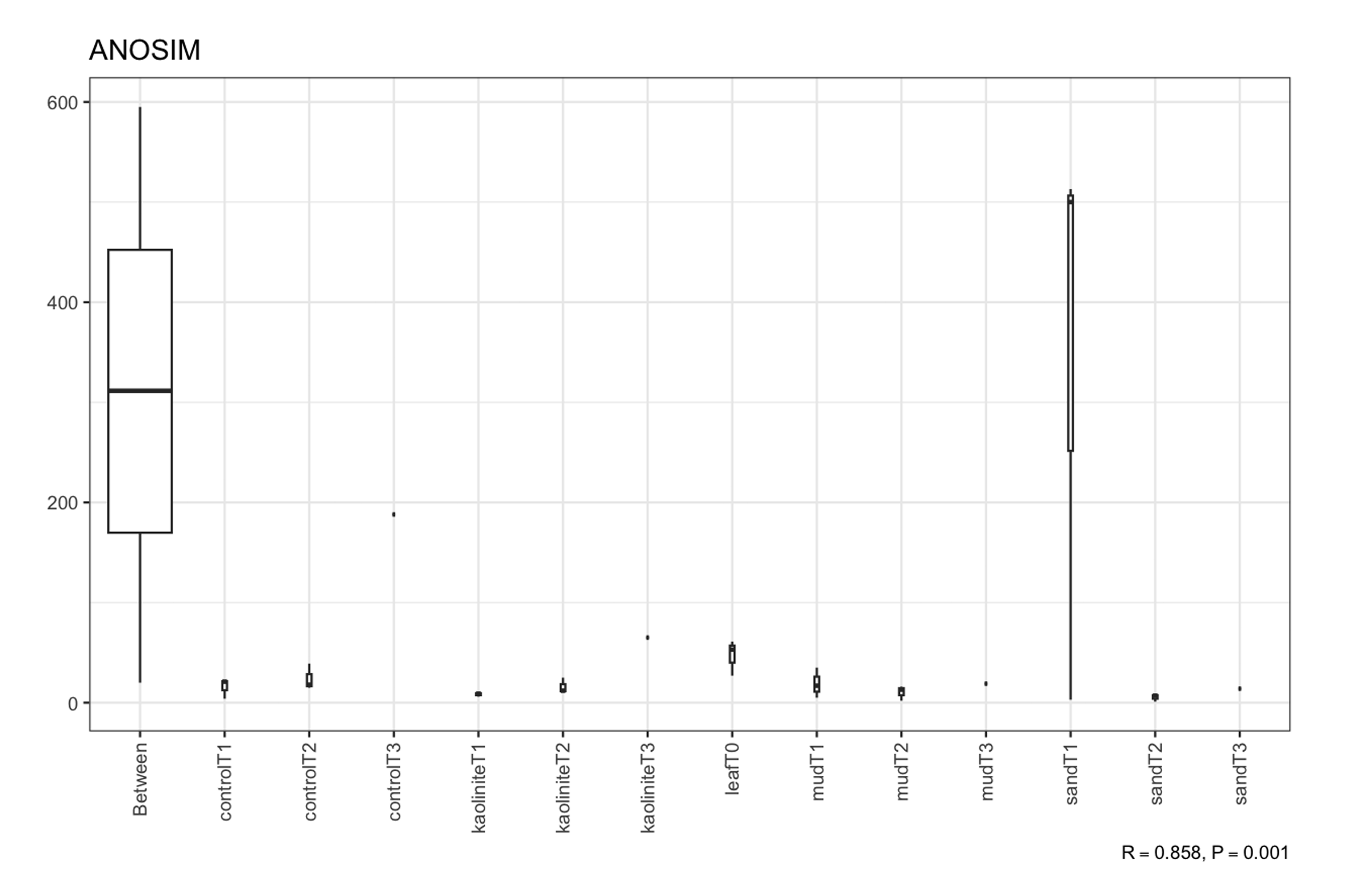
**

**Fig. S2:** Results of the ANOSIM (analysis of similarities) for bacteria and fungi combined. The betweenness shows the difference in the percentage of dissimilarity between all samples within the groups with corresponding R and p-values. The other boxplots denote differences in the percentage of dissimilarity within the groups for each of the three replicates.
