# Supplementary material for: Decay experiments and microbial community analysis of water lily leaf biofilms: Sediment effects on leaf preservation potential
Source: PLoS One. 2024 Dec 18;19(12):e0315656. doi: 10.1371/journal.pone.0315656 (PMC11654923; doi:10.1371/journal.pone.0315656)
Supplement: S3 Fig — Each circle represents substrate, time, or water chemistry (Fe+Ca+K). The numbers within the circles show the fraction of variation explained by each. (DOCX) [file pone.0315656.s008.docx]

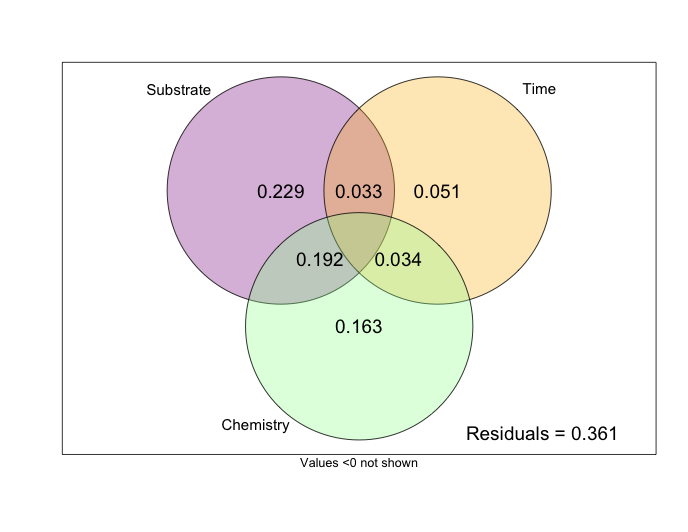


**Fig. S3:** Visualization of the results of the VPA analysis. Each circle represents substrate, time, or water chemistry (Fe+Ca+K). The numbers within the circles show the fraction of variation explained by each.
